# Supplementary material for: Multicolor interband solitons in microcombs
Source: Light Sci Appl. 2026 Mar 11;15:166. doi: 10.1038/s41377-026-02200-0 (PMC12979591; doi:10.1038/s41377-026-02200-0)
Supplement: Supplementary file 1 — Supplementary Material [file 41377_2026_2200_MOESM1_ESM.pdf]

Supplementary information for 'Multicolor interband solitons in microcombs'

Qing-Xin Ji, Hanfei Hou, Jinhao Ge, Yan Yu, Maodong Gao,
Warren Jin, Joel Guo, Lue Wu, Peng Liu,
Avi Feshali, Mario Paniccia, John Bowers, Kerry Vahala

January 5, 2026

1 Detailed Theoretical Analysis

The analysis starts with the coupled Lugiato-Lefever equations (also in the main text),

$$\begin{aligned}\frac{\partial}{\partial T}E_\mathrm{p} = -(\frac{\kappa_\mathrm{p}}{2}+i\delta\omega_\mathrm{p})E_\mathrm{p} + i\frac{D_{2,\mathrm{p}}}{2}\frac{\partial^2}{\partial\phi^2}E_\mathrm{p} + 2ig^*_\mathrm{FWM}E_\mathrm{s}E_\mathrm{i}E^*_\mathrm{p} \\ + i(g_0|E_\mathrm{p}|^2+2g_\mathrm{XPM}|E_\mathrm{s}|^2+2g_\mathrm{XPM}|E_\mathrm{i}|^2)E_\mathrm{p} + F\end{aligned} \tag{S1}$$

$$\begin{aligned}\frac{\partial}{\partial T}E_\mathrm{s} = -(\frac{\kappa_\mathrm{s}}{2}+i\delta\omega_\mathrm{s})E_\mathrm{s} - \Delta D_{1,\mathrm{s}}\frac{\partial}{\partial\phi}E_\mathrm{s} + i\frac{D_{2,\mathrm{s}}}{2}\frac{\partial^2}{\partial\phi^2}E_\mathrm{s} \\ + i(g_0|E_\mathrm{s}|^2+2g_\mathrm{XPM}|E_\mathrm{p}|^2+2g_\mathrm{XPM}|E_\mathrm{i}|^2)E_\mathrm{s} \\ + ig_\mathrm{FWM}E_\mathrm{p}^2E_\mathrm{i}^*\end{aligned} \tag{S2}$$

$$\begin{aligned}\frac{\partial}{\partial T}E_\mathrm{i} = -(\frac{\kappa_\mathrm{i}}{2}+i\delta\omega_\mathrm{i})E_\mathrm{i} - \Delta D_{1,\mathrm{i}}\frac{\partial}{\partial\phi}E_\mathrm{i} + i\frac{D_{2,\mathrm{i}}}{2}\frac{\partial^2}{\partial\phi^2}E_\mathrm{i} \\ + i(g_0|E_\mathrm{i}|^2+2g_\mathrm{XPM}|E_\mathrm{p}|^2+2g_\mathrm{XPM}|E_\mathrm{s}|^2)E_\mathrm{i} \\ + ig_\mathrm{FWM}E_\mathrm{p}^2E_\mathrm{s}^*\end{aligned} \tag{S3}$$

We take an approximate ansatz for the electric fields of the three components,

$$E_\mathrm{p} = A_\mathrm{p}\mathrm{sech}(B\phi), \tag{S4}$$

$$E_\mathrm{s} = A_\mathrm{s}\mathrm{sech}^\gamma(B\phi)e^{-i\Delta\mu_\mathrm{s}\phi}, \tag{S5}$$

$$E_\mathrm{i} = A_\mathrm{i}. \tag{S6}$$

Under constant-pump approximation $|A_\mathrm{s}|,|A_\mathrm{i}| \ll |A_\mathrm{p}|$, the primary soliton takes the unperturbed form

$$A_\mathrm{p} = \sqrt{\frac{2\delta\omega_\mathrm{p}}{g_0}}e^{i\theta_\mathrm{p}}, \tag{S7}$$

$$B = \sqrt{\frac{2\delta\omega_\mathrm{p}}{D_{2,\mathrm{p}}}}. \tag{S8}$$

And by neglecting the higher-order terms of E_s and E_i, the dynamics of E_s and E_i reads

$$\begin{aligned}\frac{\partial}{\partial T}E_\mathrm{s} = -(\frac{\kappa_\mathrm{s}}{2}+i\delta\omega_\mathrm{s})E_\mathrm{s} - \Delta D_{1,\mathrm{s}}\frac{\partial}{\partial\phi}E_\mathrm{s} + i\frac{D_{2,\mathrm{s}}}{2}\frac{\partial^2}{\partial\phi^2}E_\mathrm{s} \\ + ig_\mathrm{FWM}E_\mathrm{p}^2E_\mathrm{i}^* + 2ig_\mathrm{XPM}|E_\mathrm{p}|^2E_\mathrm{s},\end{aligned} \tag{S9}$$

$$\begin{aligned}\frac{\partial}{\partial T}E_\mathrm{i} = -(\frac{\kappa_\mathrm{i}}{2}+i\delta\omega_\mathrm{i})E_\mathrm{i} - \Delta D_{1,\mathrm{i}}\frac{\partial}{\partial\phi}E_\mathrm{i} + i\frac{D_{2,\mathrm{i}}}{2}\frac{\partial^2}{\partial\phi^2}E_\mathrm{i} \\ + ig_\mathrm{FWM}E_\mathrm{p}^2E_\mathrm{s}^* + 2ig_\mathrm{XPM}|E_\mathrm{p}|^2E_\mathrm{i}.\end{aligned} \tag{S10}$$

For stable mode-locking of the secondary soliton, we first consider the imaginary part of its dynamics eqn. (S9). From eqn. (S5), it is calculated

$$\frac{\partial}{\partial \phi} E_{\mathrm{s}} = -(\gamma B \tanh(B\phi) + i\Delta\mu_{\mathrm{s}}) E_{\mathrm{s}} \tag{S11}$$

$$\frac{\partial^2}{\partial \phi^2} E_{\mathrm{s}} = (\gamma^2 B^2 - \Delta\mu_{\mathrm{s}}^2 + 2i\gamma B \Delta\mu_{\mathrm{s}} \tanh(B\phi) - \gamma(1+\gamma) B^2 \mathrm{sech}^2(B\phi)) E_{\mathrm{s}} \tag{S12}$$

Inserting eqn. (S11)(S12) into eqn. (S9), we see it is forced that

$$\Delta\mu_{\mathrm{s}} = \frac{\Delta D_{1,\mathrm{s}}}{D_{2,\mathrm{s}}} \tag{S13}$$

$$\gamma(1+\gamma) = \frac{4 g_{\mathrm{XPM}} |A_{\mathrm{p}}|^2}{B^2 D_{2,\mathrm{s}}} = \frac{4 g_{\mathrm{XPM}}}{g_0} \frac{D_{2,\mathrm{p}}}{D_{2,\mathrm{s}}}. \tag{S14}$$

This leads to a central frequency shift for the secondary soliton, whose central angular frequency would be

$$\omega_{\mathrm{s,center}} = \omega_{\mathrm{s}} - \frac{\Delta D_{1,\mathrm{s}}}{D_{2,\mathrm{s}}} D_{1,\mathrm{p}} \tag{S15}$$

In the following, for simplicity we set $\Delta D_{1,\mathrm{s}} = 0$ to derive the threshold condition. For eqn. (S9), by multiplying by $\mathrm{sech}(B\phi)^\gamma$, and integrating over the cavity coordinate from 0 to 2π, it is derived

$$\frac{\partial}{\partial T} A_{\mathrm{s}} = -(\frac{\kappa_{\mathrm{s}}}{2} + i(\delta\omega_{\mathrm{s}} - \gamma^2 \frac{D_{2,\mathrm{s}}}{D_{2,\mathrm{p}}} \delta\omega_{\mathrm{p}})) A_{\mathrm{s}} + \frac{2i g_{\mathrm{FWM}} \delta\omega_{\mathrm{p}}}{g_0} e^{2i\theta_{\mathrm{p}}} \frac{\Pi(\gamma+2)}{\Pi(2\gamma)} A_{\mathrm{i}}^*. \tag{S16}$$

Similarly, for eqn. (S10), by integrating over the cavity coordinate from 0 to 2π, it is derived

$$\begin{aligned} \frac{\partial}{\partial T} A_{\mathrm{i}}^* = &-(\frac{\kappa_{\mathrm{i}}}{2} - i(\delta\omega_{\mathrm{i}} - \frac{2 g_{\mathrm{XPM}} \sqrt{2 D_{2,\mathrm{p}} \delta\omega_{\mathrm{p}}}}{\pi g_0})) A_{\mathrm{i}}^* \\ &- \frac{i g_{\mathrm{FWM}}^* \sqrt{D_{2,\mathrm{p}} \delta\omega_{\mathrm{p}}}}{\sqrt{2} \pi g_0} e^{-2i\theta_{\mathrm{p}}} A_{\mathrm{s}} \Pi(\gamma+2), \end{aligned} \tag{S17}$$

where $\Pi(t) \equiv \int_{-\infty}^{\infty} \mathrm{sech}^t x dx$. The dynamics now reduces to a set of linear ordinary differential equations. Its eigenvalue λ is given by

$$\begin{aligned} &(\lambda + \frac{\kappa_{\mathrm{s}}}{2} + i(\delta\omega_{\mathrm{s}} - \gamma^2 \frac{D_{2,\mathrm{s}}}{D_{2,\mathrm{p}}} \delta\omega_{\mathrm{p}}))(\lambda + \frac{\kappa_{\mathrm{i}}}{2} - i(\delta\omega_{\mathrm{i}} \\ &- \frac{2 g_{\mathrm{XPM}} \sqrt{2 D_{2,\mathrm{p}} \delta\omega_{\mathrm{p}}}}{\pi g_0})) - \frac{2 |g_{\mathrm{FWM}}|^2 \delta\omega_{\mathrm{p}}^2}{\pi g_0^2} \sqrt{\frac{D_{2,\mathrm{p}}}{2\delta\omega_{\mathrm{p}}}} \frac{\Pi(\gamma+2)^2}{\Pi(2\gamma)} = 0, \end{aligned} \tag{S18}$$

A threshold behaviour is hence predicted. The secondary soliton emerges only when A_{s} experiences exponential growth. Threshold is reached when an eigenvalue is purely imaginary. The threshold condition is thus

$$\begin{aligned} &\frac{\kappa_{\mathrm{s}} \kappa_{\mathrm{i}}}{4} + (\frac{\delta\omega_{\mathrm{s}} + \delta\omega_{\mathrm{i}}}{2} - \frac{g_{\mathrm{XPM}} \sqrt{2 D_{2,\mathrm{p}} \delta\omega_{\mathrm{p}}}}{\pi g_0} - \gamma^2 \frac{D_{2,\mathrm{s}}}{2 D_{2,\mathrm{p}}} \delta\omega_{\mathrm{p}})^2 \\ &- \frac{2 |g_{\mathrm{FWM}}|^2 \delta\omega_{\mathrm{p}}^2}{\pi g_0^2} \sqrt{\frac{D_{2,\mathrm{p}}}{2\delta\omega_{\mathrm{p}}}} \frac{\Pi(\gamma+2)^2}{\Pi(2\gamma)} = 0. \end{aligned} \tag{S19}$$

2 Calculation of Nonlinear Coefficients

While the nonlinear coefficient g in the Lugiato-Lefever equation for a single cavity has an established expression [1],

$$g = \frac{\hbar \omega_{\mathrm{p}}^2 c n_2}{n_{\mathrm{g}}^2 V_{\mathrm{eff}}} = \frac{\hbar \omega_{\mathrm{p}}^2 D_{1,\mathrm{p}} n_2}{2\pi n_{\mathrm{g}} A_{\mathrm{eff}}} \tag{S20}$$

where n_2 is the nonlinear refractive index, n_g is the group index and A_{eff} is the effective mode area, the coefficients g_0, g_{XPM} and g_{FWM} in coupled LLEs (S1)-(S3) for the coupled rings system have not yet been defined. In this section a link between these coefficients and the single-cavity coefficient g is derived.

Generally, in a microresonator, for a third-order nonlinear process denoted as $\omega_{\text{j}} + \omega_{\text{k}} \to \omega_{\text{l}} + \omega_{\text{m}}$, its nonlinear coefficient g_{jklm} is expressed as [2]

$$g_{jklm} = \frac{\hbar\sqrt{\omega_j\omega_k\omega_l\omega_m}cn_2}{n_{\text{g}}^2}\frac{\int F_j^* F_k^* F_l F_m dV}{\sqrt{\int|F_j|^2dV\int|F_k|^2dV\int|F_l|^2dV\int|F_m|^2dV}} \tag{S21}$$

Here, $F(x,y,z)$ denotes the field amplitude distribution. For a single cavity, this is readily accessible from mode simulation. Instead, in coupled rings, the cavity modes hybridize to form three supermode families [1]. For all supermode families, the field distributions inside ring A, B, C share the same pattern $F_0(x,y,z)$ (Fig. S1a,b), but the intensity is different across rings. It can be written that

$$F_{Jk}(x,y,z) = c_{Jk}F_0(x,y,z) \tag{S22}$$

where $J = \text{A},\text{B},\text{C}$ stands for different rings, $k = \text{p},\text{s},\text{i}$ stands for pump, signal and idler frequencies. Amplitudes c_{Jk} are normalized, that

$$|c_{\text{A}k}|^2+|c_{\text{B}k}|^2+|c_{\text{C}k}|^2 = 1 \tag{S23}$$

The nonlinear coefficients in our case can be derived based on above analysis. Take the self-phase modulation coefficient at pump band g_{p} for example,

$$g_{\text{p}} = \frac{\hbar\omega_{\text{p}}^2cn_2}{n_{\text{g}}^2}\frac{\int_{\text{A}}|F_{\text{Ap}}|^4dV + \int_{\text{B}}|F_{\text{Bp}}|^4dV + \int_{\text{C}}|F_{\text{Cp}}|^4dV}{(\int_{\text{A}}|F_{\text{Ap}}|^2dV + \int_{\text{B}}|F_{\text{Bp}}|^2dV + \int_{\text{C}}|F_{\text{Cp}}|^2dV)^2} \tag{S24}$$

$$= \frac{\hbar\omega_{\text{p}}^2cn_2}{n_{\text{g}}^2}\frac{\int|F_0|^4dV}{(\int|F_0|^2dV)^2}(|c_{\text{Ap}}|^4+|c_{\text{Bp}}|^4+|c_{\text{Cp}}|^4) \tag{S25}$$

$$= g(|c_{\text{Ap}}|^4+|c_{\text{Bp}}|^4+|c_{\text{Cp}}|^4) \tag{S26}$$

The expressions for other coefficients are (g_{j} for self-phase-modulation, $g_{\text{j,k}}$ for cross-phase-modulation, and g_{FWM} for four-wave-mixing)

$$g_{\text{s}} = g(|c_{\text{As}}|^4+|c_{\text{Bs}}|^4+|c_{\text{Cs}}|^4) \tag{S27}$$
$$g_{\text{i}} = g(|c_{\text{Ai}}|^4+|c_{\text{Bi}}|^4+|c_{\text{Ci}}|^4) \tag{S28}$$
$$g_{\text{p,s}} = g(|c_{\text{Ap}}|^2|c_{\text{As}}|^2+|c_{\text{Bp}}|^2|c_{\text{Bs}}|^2+|c_{\text{Cp}}|^2|c_{\text{Cs}}|^2) \tag{S29}$$
$$g_{\text{p,i}} = g(|c_{\text{Ap}}|^2|c_{\text{Ai}}|^2+|c_{\text{Bp}}|^2|c_{\text{Bi}}|^2+|c_{\text{Cp}}|^2|c_{\text{Ci}}|^2) \tag{S30}$$
$$g_{\text{s,i}} = g(|c_{\text{As}}|^2|c_{\text{Ai}}|^2+|c_{\text{Bs}}|^2|c_{\text{Bi}}|^2+|c_{\text{Cs}}|^2|c_{\text{Ci}}|^2) \tag{S31}$$
$$g_{\text{FWM}} = g(c_{\text{Ap}}^2c_{\text{As}}^*c_{\text{Ai}}^* + c_{\text{Bp}}^2c_{\text{Bs}}^*c_{\text{Bi}}^* + c_{\text{Cp}}^2c_{\text{Cs}}^*c_{\text{Ci}}^*) \tag{S32}$$

The next step is to determine amplitudes c_{Jk}. To this end, the transfer matrix formalism in [1] is adopted to calculate the eigenmodes, and the parameters are fitted by measuring the eigenmode spectrum.

In Fig. S1c, measured dispersion data and its fitting result are presented (also in Fig. 1g in the main text). Fitted parameters are $g_{\text{co}}L_{\text{co}} = 0.878$, $\mu_g = 1110$, $\epsilon_1 = 2.86 \times 10^{-3}$, $\phi_2 = 0.411$ (see definition in [1]). With fitted parameters, the eigenvectors are solved, and the energy distribution between three rings of different transverse modes are plotted in Fig. S1d. The pump and signal, idler sidebands are located at $\mu = 0, 61, -61$, respectively. The amplitudes involved in (S26)-(S32) read $c_{\text{Ap}} = 0.2699 - 0.1848i$, $c_{\text{Bp}} = 0.6862$, $c_{\text{Cp}} = -0.5686 - 0.3144i$, $c_{\text{As}} = 0.7028$, $c_{\text{Bs}} = -0.2221 + 0.2171i$, $c_{\text{Cs}} = -0.6367 + 0.0656i$, $c_{\text{Ai}} = 0.2059 + 0.4083i$, $c_{\text{Bi}} = 0.2385 - 0.0339i$, $c_{\text{Ci}} = 0.8561$. Finally, the nonlinear coefficients are calculated to be $g_{\text{p}} = 0.4114g$, $g_{\text{s}} = 0.4210g$, $g_{\text{i}} = 0.5842g$, $g_{\text{p,s}} = 0.2712g$, $g_{\text{p,i}} = 0.3591g$, $g_{\text{s,i}} = 0.4091g$, $g_{\text{FWM}} = (-0.1467 - 0.2610i)g$, while for a single ring, $g/2\pi = 4.33 \times 10^{-3}$ Hz is obtained from mode simulation.

In the analytical theory and simulation presented in the main text, for simplicity we have ignored the difference in nonlinear coefficients at different dispersion bands and assume that $g_{\text{p}} = g_{\text{s}} = g_{\text{i}} = g_0$, $g_{\text{p,s}} = g_{\text{p,i}} = g_{\text{s,i}} = g_{\text{XPM}}$. The coefficient values used in simulation are on the same order as above values.

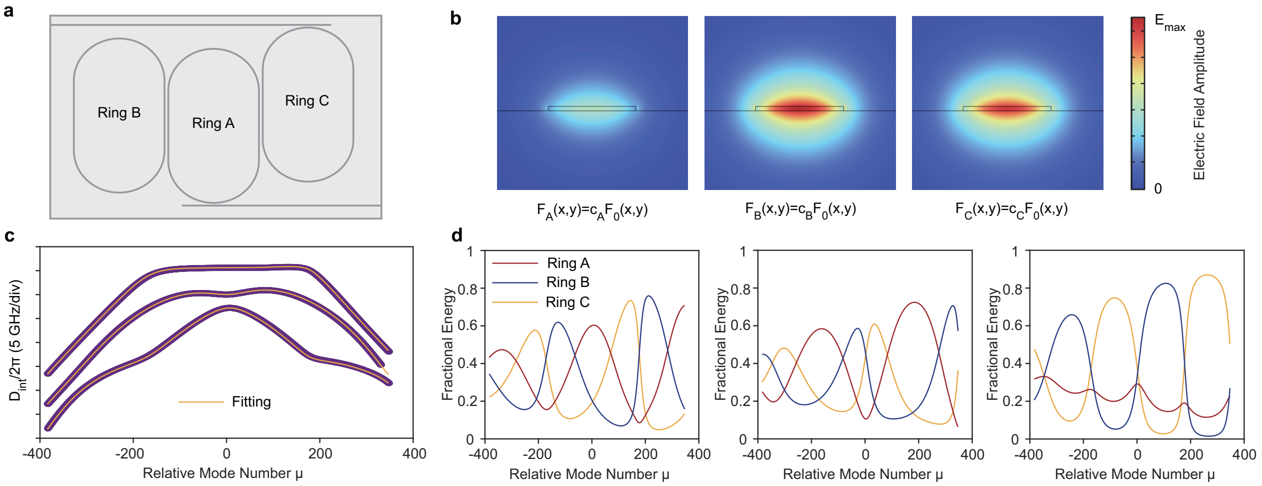

Fig. S1: **Supermodes of three-coupled-ring microresonator. a,** Layout of ring A, B and C. **b,** Transverse mode profile of the pumped mode. Fundamental TE mode is hybridized, where the normalized amplitudes are $|c_{\mathrm{A}}| = 0.3271$, $|c_{\mathrm{B}}| = 0.6862$, $|c_{\mathrm{C}}| = 0.6497$. **c,** Fitting result of measured dispersion data. **d,** Modal energy distribution across the three rings at three dispersion bands. From left to right, upper, middle and lower dispersion band.

3 Essentiality of cross-phase-modulation effect

Cross-phase-modulation (XPM) effect is essential to multicolor interband solitons formation. We verify by simulation that disabling XPM effect suppresses stable secondary-soliton formation, while re-enabling XPM restores it.

In Fig. S2, steady-state time-domain waveforms with and without XPM are shown. For Fig. S2a, the same simulation parameters are used as in Fig. 4a in the main text, while for Fig. S2b, the XPM nonlinear coefficient g_{XPM} is set to 0, and other parameters are kept unchanged. Without XPM effect, although parametric oscillation occurs, the parametric sideband cannot reach stable soliton-mode-locking.

References

[1] Yuan, Z. *et al.* Soliton pulse pairs at multiple colours in normal dispersion microresonators. *Nature Photonics* **17**, 977–983 (2023).

[2] Agrawal, G. Chapter 10 - four-wave mixing. In Agrawal, G. (ed.) *Nonlinear Fiber Optics (Fifth Edition)*, Optics and Photonics, 397–456 (Academic Press, Boston, 2013), fifth edition edn.

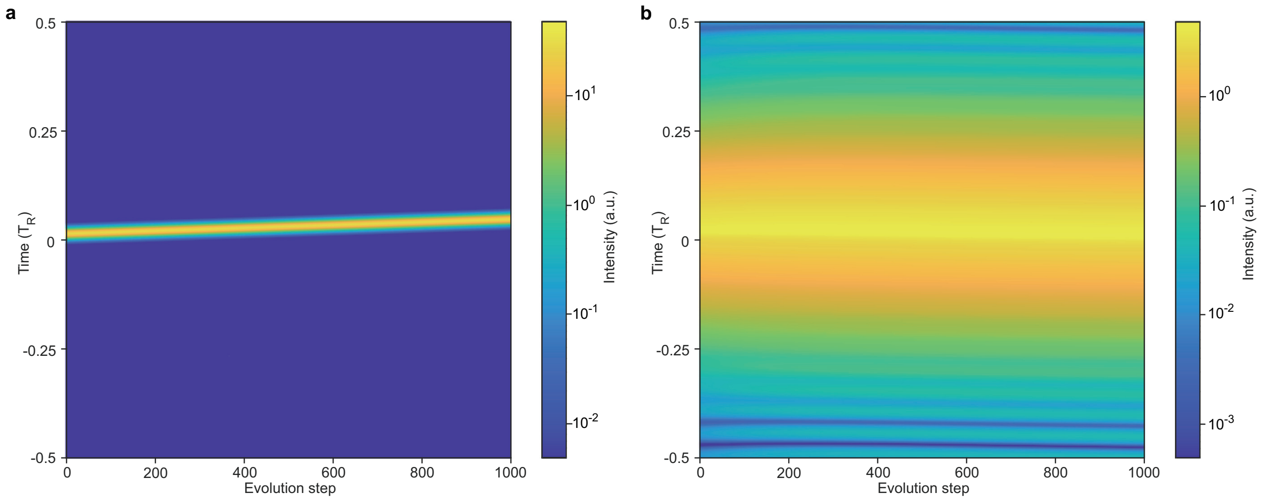

Fig. S2: **Comparison of steady-state time-domain waveform evolution with and without XPM.** **a,** Waveform evolution with XPM, featuring mode-locked secondary soliton. **b,** Waveform evolution without XPM, where the signal sideband is non-mode-locked. T_{R} is the round-trip time.
